# Efficacy of vitamin C supplementation as an adjunct in the non-surgical management of periodontitis: a systematic review

**DOI:** 10.1186/s13643-020-01554-9

**Published:** 2021-01-04

**Authors:** Hytham N. Fageeh, Hammam I. Fageeh, Ashwin Prabhu, Shilpa Bhandi, Shahrukh Khan, Shankargouda Patil

**Affiliations:** 1 Department of Preventive Dental Sciences, College of Dentistry, Jazan University, Jazan, Kingdom of Saudi Arabia; 2 Department of Periodontics, KLE Society’s Institute of Dental Sciences, Bangalore, India; 3 Department of Restorative Sciences, College of Dentistry, Jazan University, Jazan, Kingdom of Saudi Arabia; 4 School of Nursing and Midwifery, Faculty of Health, Deakin University, Geelong, Australia; 5 Department of Maxillofacial Surgery & Diagnostic Sciences, Division of Oral Pathology, College of Dentistry, Jazan University, Jazan, Kingdom of Saudi Arabia

**Keywords:** Vitamin C, Ascorbic acid, Periodontal diseases, Gingivitis, Periodontitis

## Abstract

**Background and objective:**

The antioxidant potential of vitamin C is useful in reducing oxidative stress, free radicals, and reactive oxygen species, which may assist in the improved outcomes of periodontal therapy. This systematic review was aimed to evaluate the effectiveness of vitamin C supplementation as an adjunct to non-surgical periodontal therapy, in the management of periodontitis.

**Data sources:**

PubMed, EMBASE, Cochrane Library, and Web of Science.

**Study eligibility criteria:**

Randomized controlled trials published between January 1990 and March 2020.

**Participants:**

People 18 years and older with periodontitis.

**Study appraisal and synthesis methods:**

The Critical Appraisal Skills Programme (CASP) quality appraisal tool.

**Results:**

The initial search yielded 441 articles out of which six studies fulfilled the inclusion criteria. Vitamin C supplementation helped improve bleeding indices in gingivitis but did not significantly lead to reduction of probing depths or clinical attachment gain for periodontitis.

**Conclusion:**

Administration of vitamin C as an adjunct to non-surgical periodontal therapy did not result in clinically significant improvements in pocket probing depths at 3 months in periodontitis patients. With the limited evidence available, no recommendation can be made for supplementation of vitamin C in conjunction with initial periodontal therapy for subjects with periodontitis to improve primary treatment outcome measures.

## Clinical relevance

### Rationale

- The appropriate supplementation of vitamin C may lead to lower levels of inflammation owing to its antioxidant properties. If used in conjunction with non-surgical periodontal therapy, it may lead to better treatment outcomes. Since periodontal diseases burden global healthcare systems, it is imperative to find cost-effective approaches to prevent or slow its progression.

### Principal findings

- Supplemental use of vitamin C as an adjunct to non-surgical therapy did not result in a clinically significant improvement in pocket probing depths at 3 months in periodontitis patients, whereas in gingivitis and in diabetic individuals, supplementation showed improvement in gingival parameters of bleeding and inflammation.

### Practical implications

- Inclusion of vitamin C supplements in non-surgical treatment protocols may not offer any additional benefit clinically in improving treatment outcomes for patients with periodontitis.

## Background

Periodontitis is a multifactorial, immuno-inflammatory disease primarily initiated by an infection of bacteria from the dental biofilm, followed by an anomalous host response, leading to the destruction of periodontal tissues [1]. Periodontitis is a complex condition that is not fully understood despite significant advances in unraveling the underlying disease mechanisms. Current understanding attributes a major role in the various stages of periodontitis to neutrophils and their action [2].

Polymorphonuclear leukocytes (PMNs) are known to play a critical role in the pathobiology of periodontitis by mounting an antimicrobial response to biofilm bacteria. Infiltration of periodontal tissue by PMNs leads to multiple signaling pathways being triggered and increasing reactive oxygen species (ROS) concentration at the site of infection [3].

ROS is a broad term that includes oxygen-derived free radicals, nitric oxide radical species, and non-radical derivatives of oxygen which are essential to many normally occurring biologic processes. The human body has also developed a highly integrated and complex antioxidant defense system that functions to detoxify ROS and modifying them to form less reactive species thereby nullifying their biologic effects. It has been found that ROS, in cultures, demonstrates a growth promotive effect on fibroblasts and epithelial cells at low concentrations. However, at higher concentrations, they have a detrimental effect culminating in tissue damage in the absence or dysfunction of antioxidant mechanisms [4]. In the context of periodontal diseases, ROS along with inflammatory mediators and lipid peroxides together activate macrophages, fibroblasts, and more neutrophils leading to overproduction of ROS resulting in increased “oxidative stress” and a vicious cycle results from it [5]. This “oxidative stress” brought about by PMNs is suggested to be primarily responsible for the destruction of tissues observed in periodontitis [6].

Vitamin C or l-ascorbic acid is a compound that belongs to the scavenging (chain breaking) group of antioxidants [7]. It is known to play a vital role in the maintenance of the integrity of connective tissue, osteoid tissues, and dentine apart from its activity as an enzyme cofactor in its ionic form as ascorbate [7]. It also scavenges free radicals and possesses potent antioxidant and immune-modulatory properties which can effectively control excessive ROS generated in many chronic inflammatory conditions [8]. Vitamin C is also considered an important dietary antioxidant in the maintenance of periodontal health [5]. Furthermore, it has been shown to decelerate the rate of progression of periodontitis by stimulating the differentiation of progenitor cells of the periodontal ligament [9].

Many experimental and epidemiological studies have been undertaken to explore the preventative and therapeutic potential of vitamin C concerning periodontal diseases. While there is insufficient evidence to support the use of antioxidant vitamins as a monotherapy in the management of periodontal diseases [10, 11], its role as a preventive agent and as an adjunct to non-surgical treatment cannot be dismissed. Indeed, experimental gingivitis studies have shown that the intake of ascorbic acid either by diet or supplementation has a strong preventive effect on the reduction of gingival bleeding [11]. Our present understanding of periodontal diseases also suggests that gingivitis and periodontitis may present as individual pathologies and, therefore, respond differently to the benefits of vitamin C supplementation [12]. Despite this relationship and given the limited number of controlled studies, it has been difficult to ascertain the effect of vitamin C supplementation on clinical parameters used to assess periodontal treatment outcomes [12].

Thus, this systematic review was undertaken to evaluate the adjunctive effects of dietary supplementation of vitamin C on clinical parameters such as periodontal pocket depth (PPD) and clinical attachment level (CAL) in conjunction with non-surgical periodontal treatment (NSPT) in patients with periodontitis.

## Methodology

### Protocol development

The systematic review was registered in PROSPERO with the registration number CRD42020179309 and the PRISMA (Preferred Reporting Items for Systematic Reviews and Meta-Analyses) statement checklist for reporting was adopted [13]. This review focused on studies that investigated the effect of vitamin C supplementation as an adjunct to non-surgical periodontal therapy and its impact on PPD and clinical attachment level [CAL].

### Eligibility criteria

The criteria used for this article was according to the PICOS format: The population (P) included adult participants that were non-institutionalized with periodontitis (notwithstanding the definition, extent, and severity of the periodontitis) and no systemic comorbidities or other specialist orthodontic treatment; the intervention (I) was the dietary supplementation of vitamin C; the comparison (C) was among the periodontitis patients with and without the vitamin C dietary intake; the outcome (O) was the status of the periodontitis evaluated by several parameters including dental plaque index (PI), PPD, clinical attachment level (CAL), bleeding on probing (BOP), gingival index (GI), gingivitis severity index (GSI), and sulcus bleeding index (SBI); and the study design (S) included randomized control trials (RCTs).

### Information sources and search

MEDLINE (through PubMed), EMBASE, Cochrane Oral Health Group Specialized Register, Google Scholar, ProQuest Dissertations, and Thesis Database were used in this systematic review. The following search terms were used: (Vitamin C) OR (Ascorbic Acid) AND (Periodontal Disease) OR (Periodontitis) OR (Dental plaque) OR (Gingival bleeding) OR (Bleeding on Probing) OR (BOP) OR (Periodontal Pocket) OR (Periodontal Probing Depth) OR (Clinical Attachment Loss).

### Study selection and data extraction

The articles were screened by two reviewers (HNF and HIF) who independently checked for relevant titles and abstracts from articles published until April 2020. Any disagreement was resolved by consensus and discussion. Both authors then performed a full-text evaluation following the PICOS criteria. A Microsoft Excel spreadsheet was created to summarize the findings from the full-text analyses.

### Risk of bias

After the full-text assessment was done, both reviewers started data extraction and risk of bias evaluation, which was assessed by using appropriate tools according to study design. The revised Cochrane risk of bias tool for randomized trials (RoB-2) was used [14].

### Summary measures and synthesis of results

For both test and control groups, mean values and standard deviation (SD) were evaluated in the studies selected. For the size of the intervention effect in the individual studies, the standardized mean difference (SMD), and 95% confidence interval (CI) were analyzed as the difference in the mean outcome between groups. The cumulative effect was considered significant if *p* < 0.05.

### Quality appraisal of included studies

The Critical Appraisal Skills Programme (CASP) guidelines checklist was used for the appraisal of randomized controlled trials in this review [15]. CASP quality assessment is based on nine questions that ask if the study focused issue, randomized assignment of patients, did the proper selection of patients, blinded experiment, identified similarity of the groups at the beginning of the trial, treated the groups equally, applied results in the context, considered clinically important outcomes, and weighted for benefits over harms and costs. The authors (H.N.F. and S.P.) independently graded each included study using the CASP quality assessment criteria for randomized controlled trials and then resolved the potential conflict with an independent oral health research expert. The CASP was graded as “High”, “Moderate”, and “Low” based on the strengths and weaknesses of studies. The tool generates binary scores: 1 for “satisfied” and 0 for “unsatisfied”.

## Results

### Study selection

**Fig. 1:**
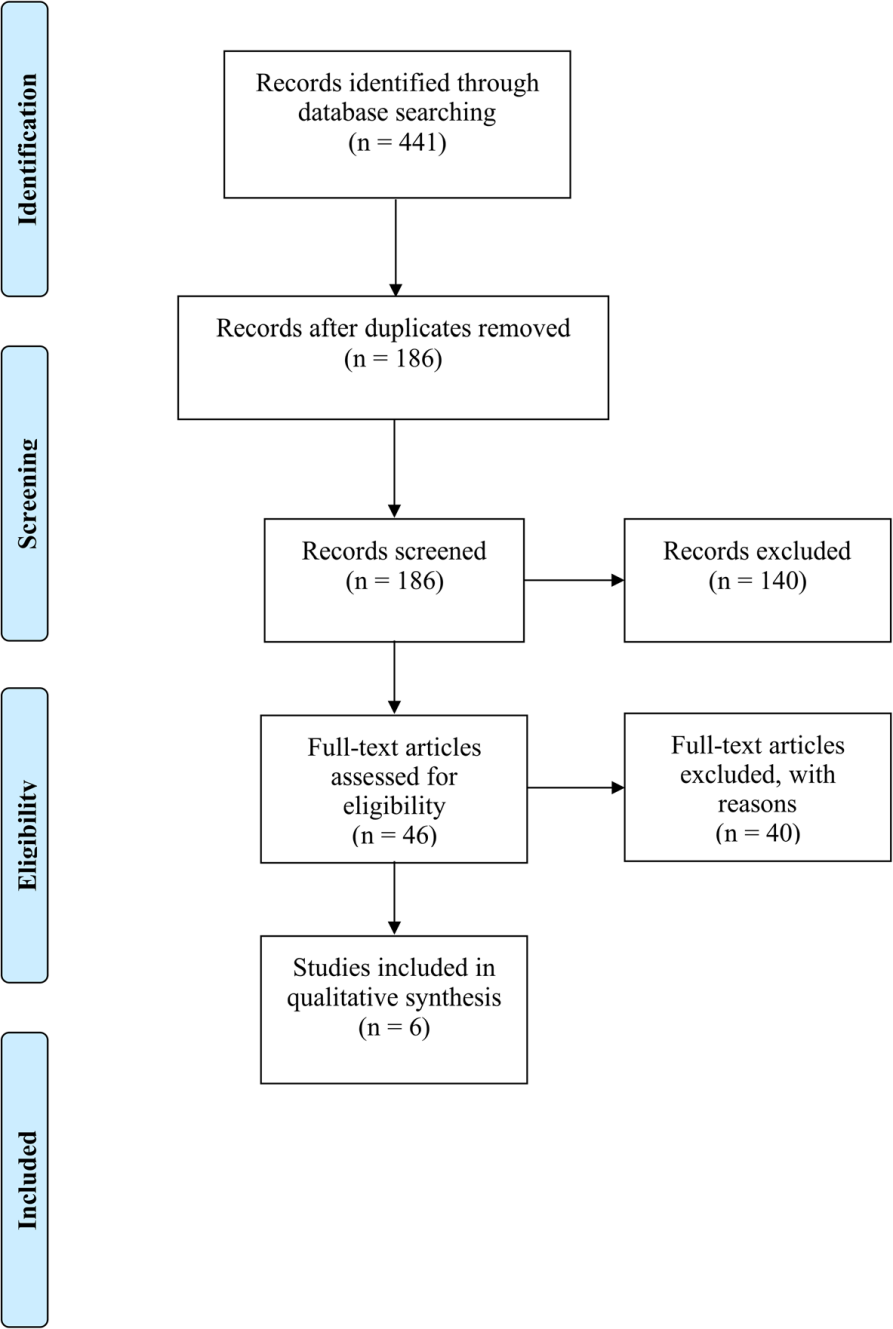
The PRISMA flowchart that was used for this systematic review

The search yielded 441 articles, of which 255 duplicates were removed. The remaining 186 articles were screened using their titles and abstract. Of these studies, 140 were further excluded, leaving 46 articles for full-text analysis. The final list of selected articles included six relevant articles [16–21]. The PRISMA flowchart in Fig. 1 shows the selection process. The inter-examiner agreement was 0.80 for the title and abstract screening and 0.79 for the full-text evaluation via Cohen’s kappa test [22].

### Study characteristics

Overall, the six RCTs included in this review were published from 2010 to 2019 with adult participants > 23 years of age. The minimum follow-up period ranged from 14 days to 6 months, with four studies mandating at least a 3-month follow-up [17–19, 21]. All studies except Shimabukuro et al. contained participants that possessed chronic periodontitis. Total antioxidant capacity (TAOC) was measured by Shimabukuro et al. and Abou Sulaiman and Shehadeh in saliva and serum, respectively, and did not report serum/plasma vitamin C levels. The study by Chitsazi et al. did not report either TOAC or serum/plasma vitamin C levels. Participants in all other studies were tested for plasma/serum vitamin C levels.

### Exclusion criteria of studies

Generally, patients who were pregnant or lactating, and participants who used non-steroidal anti-inflammatory drugs (NSAIDs) or antimicrobial drugs in the past 3 months before the RCTs began were excluded from the studies. Smokers, previous or current, were exempt from all studies except Dodington et al., who assessed the effect of vitamin C on periodontitis in smokers [18]. Furthermore, only two studies evaluated the comparison between chronic periodontitis and vitamin C intake in diabetic patients who were unaffected by other medical complications [16, 20].

### Vitamin C supplementation

Vitamin C was given in varying modes and doses across the six RCTs that were assessed. Kunsongkeit et al. supplemented 500 mg/day vitamin C for 30 days after scaling and root planing [16], whereas Chitsazi et al. opted for 60 mg/day for females and 75 mg/day for males along with 2 mg/day melatonin for 4 weeks [17]. Dodington et al. experimented with varied doses of vitamin C from 42 to 107 mg/day, 108 to 149 mg/day, and 151 to 241 mg/day to study its effect on smokers and non-smokers [18]. Abou Sulaiman and Shehadeh chose 2000 mg/day of vitamin C for 4 weeks [19], while Gokhale et al. preferred the dose 450 mg/day of vitamin C as chewable tablets [20]. The only study in this review that used vitamin C as a dentifrice containing l-ascorbic acid 2-phosphate magnesium salt (0.3%) was conducted by Shimabukuro et al [21].

### Interventions and outcome measures

All RCTs used NSPT as a mode of intervention with and without supplemental vitamin C in the treatment and control groups, respectively. Vitamin C serum/plasma levels were measured in all studies except those by Shimabukuro et al. and Abou Sulaiman and Shehadeh wherein they measured total antioxidant capacity (TAOC) in saliva and plasma, respectively, and did not report serum/plasma vitamin C levels [19, 21]. Gokhale et al. tested similar measures with patients who possessed chronic gingivitis, chronic periodontitis, and chronic periodontitis with type 2 diabetes. Shimabukuro et al., on the other hand, used a dentifrice enriched with vitamin C on patients with gingivitis only while Abou Sulaiman and Shehadeh focused on NSPT with vitamin C in patients with chronic periodontitis.

The periodontal measures that were assessed included the dental plaque index (PI), probing pocket depth (PPD), clinical attachment level (CAL), bleeding on probing (BOP), gingival index (GI), gingivitis severity index (GSI), and sulcus bleeding index (SBI). All studies had examiner calibration exercise to make sure intra-examiner reliability and inter-examiner reliability before periodontal assessment. The dental professionals who performed the oral examination varied across studies based on the examiners [dental hygienist, periodontitis, or a general dentist].

### Covariates

Since Kunsongkeit et al. studied the effect of vitamin C as an adjunct in NSPT in uncontrolled type 2 diabetes mellitus patients, fasting blood sugar levels and HbA1c acted as covariates [16]. Dodington et al. tested smoking status, beta-carotene, alpha-tocopherol, vitamin D, alpha-linolenic acid (ALA), eicosapentaenoic acid (EPA), and docosahexaenoic acid (DHA) along with plasma ascorbic acid concentration [18], whereas Shimabukuro et al. tested the total antioxidant activity of the saliva [21].

### Effect of vitamin C on gingivitis and periodontitis

Five out of six included studies found that vitamin C supplementation was associated with reduction periodontal outcome measures of PI, SBI, GI, PPD, and CAL. Shimabukuro et al.’s 2015 study found that dentifrice containing l-ascorbic acid 2-phosphate magnesium salt proved beneficial in GI reduction in the test group from 1.22 ± 0.03 to 0.73 ± 0.03 and the GSI from 1.09 ± 0.04 to 0.69 ± 0.03. Chitsazi et al. study found that vitamin C with melatonin may be beneficial as an adjunct to NSPT in patients with chronic periodontitis [17]. Dodington et al. showed that a dose-response relationship was observed between vitamin C intake and reduction in sites with PPD > 3 mm [18]. Gokhale et al. and Shimabukuro et al. reported that vitamin C had a pronounced effect on patients with chronic gingivitis, although the latter only utilized a dentifrice to achieve this effect [20, 21]. Abou Sulaiman and Shehadeh reported no significant differences in periodontal condition after vitamin C supplementation as the control and the test groups exhibited similar outcomes [16, 19].

### Quality appraisal of included studies

The CASP checklist guidelines were used to assess the quality of the included studies. Table 1 shows the strengths and weaknesses of each study and Table 2 shows the study participants’ characteristics, case definition of periodontitis, intervention, and summary of result. Overall, outcomes were noted in all RCTs to minimize bias (*n* = 6; 95%). Serum/plasma levels of vitamin C were assessed in two studies only [19, 20]. The third study used GI and GSI to monitor outcomes [21].

**Table 1:** The CASP quality assessment scale (*N* = 6)

Author	Part 1	Part 2	Part 3	Part 4	Part 5	Part 6	Part 7	Part 8	Part 9	Quality
Abou Sulaiman and Shehadeh [19], Syria	1	1	1	1	1	1	1	1	1	High
Shimabukuro et al. [21], Japan	1	1	1	1	1	1	1	1	1	High
Gokhale et al. [20], India	1	1	1	1	1	1	1	1	1	High
Kunsongkeit et al. [16], Thailand	1	1	1	1	1	1	1	1	1	High
Chitsazi et al. [17], Iran	1	1	1	1	1	1	1	1	1	High
Dodington et al. [18], Canada	1	1	0	0	0	1	1	1	1	Moderate

**Table 2:** Study and participants characteristics, case definition of periodontitis, intervention, and summary of result (*N* = 6)

Author, year, country	Study design, follow-up period	Sample size calculation, random allocation, examiner calibration	Participant characteristics: age range, gender, mean age, ethics approval; pre-treatment instruction	Periodontitis definition, probe used, professional who performed SRP	Covariates and dropout	Intervention (Test)	Intervention (Control)	Summary of result
Kunsongkeit et al., 2019 [16], Thailand	RCT-double-blind, 1 and 2 month	Yes, yes, NR	31 uncontrolled type 2 diabetes mellitus and periodontitis patients; all participants received OHI + motivation until PI was ≤ 0.5 pre-treatment	PI, GI, SBI, PD, CAL; UNC 15-probe	Plasma vitamin C, FBS, HbA1c; no dropout	500 mg/day vitamin C [30 days] + FMSRP	FMSRP + placebo	The periodontal parameters of PI, SBI, GI, PD, and CAL significantly improved in the treatment group and the placebo group. No significant differences were observed in periodontal parameters in treatment group and the placebo group.
Chitsazi et al., 2017 [17], Iran	RCT, single masked; 3 and 6 month	Yes, yes, NR	60 periodontitis patients, 23-65 years, mean age 41 years, three groups [20 pts each], all participants were given OHI to toothbrush with modified bass technique + flossing twice a day	GI, PD, CAL; UNC 15-probe; periodontist	Age, sex; no dropout	Treatment group 1, NSPT + melatonin + vitamin C, treatment group 2, and NSPT + melatonin. All groups received NSPT using ultrasonic scaler and curettage. Melatonin dose, 2 mg/day for 4 weeks, and vitamin C dose, 60 mg/day for females and 75 mg/day for males for 4 weeks	NSPT only	Significant improvement in PD and CAL scores at 6-month interval compared to 3 months in the melatonin+ vitamin C group (*P* < 0.05), while the differences in PD and CAL scores between the mentioned intervals were not significant between the control and melatonin groups (*P* > 0.05).
Dodington et al., 2015 [18], Canada	RCT, NR; 3, 4 month	Yes, yes, yes	98 chronic generalized periodontitis patients [PD of 4 mm or greater in at least 30% of probed sites], mean age 59 years for non-smokers, 53 years for smokers	PD, BoP, % of sites with PD > 3 mm, electronic probe, two oral hygienists	FFQ, age, sex, health condition, medication used, smoking status, beta-carotene, alpha-tocopherol, vitamin D, alpha-linolenic acid (ALA), EPA, and DHA; 31 dropout or lost to follow-up or had missing data	NSPT in non-smokers + dietary intake recording; vitamin C intake (both treatment and control): 1. 42–107 mg/day 2. 108–149 mg/day 3. 151–241 mg/day	NSPT in smokers	A dose-response relationship was observed between vitamin C [dietary and total] intake and reduction in % of sites with PD > 3 mm in people who underwent NSPT. Fruits and vegetables, beta-carotene, alpha-tocopherol, EPA, and DHA are associated with reduced PD after SRP in non-smokers, but not smokers, with chronic generalized periodontitis.
Shimabukuro et al., 2015 [21], Japan	RCT, double-blind; 3 month	Yes, yes, yes	300 participants [150 test, 150 control], 20–64 years	GI, GSI	Gingival redness, gingival severity index, total antioxidant activity (TAOC) of the saliva	Dentifrice containing l-ascorbic acid 2-phosphate magnesium salt [0.3%]	Dentifrice without APM	GI, gingival redness, and GSI significantly reduced in the intervention group as compared to the control group. The result suggested that dentifrice containing ascorbic acid were effective in reducing gingivitis.
Gokhale et al., 2013 [23], India	RCT, double blinded; 14 days	NR, NR, NR	121 participants with SBI ≥ 2, 30–60 years, four groups: 30 healthy without periodontitis, 30 healthy with periodontitis, 30 healthy with chronic gingivitis, and 30 diabetics with periodontitis	PI, PPD, SBI; periodontitis was defined based on Armitage 1999 definition; chronic gingivitis was defined based on SBI; single dentist	Plasma ascorbic acid concentration	Group1: SRP over two appointment + ascorbic acid supplementation [450 mg] as chewable tablet	SRP over two appointment + lemon flavored chewable tablet	Vitamin C supplementation as an adjunct to NSPT significantly improved the SBI in participants with gingivitis, and diabetics with periodontitis.
Abou Sulaiman and Shehadeh, 2010 [19], Syria	RCT, single masked; 1 and 3 months	Yes, yes	60 chronic periodontitis patients [30 treatment group and 30 control group]	PPD, CAL, BOP, GI; standard periodontal probe; single specialist; periodontist	Age, sex; total antioxidant activity (TAOC) of plasma (1 month after periodontal treatment)	Group 1: NSPT + vitamin C (dose of vitamin C, 2000 mg a day for 4 weeks); group 2: NSPT with no vitamin C	NSPT only	There were significant improvements in PPD, CAL, GI, and BOP scores at the 1-month and 3-month interval post-treatment in both the ChP1 and ChP2 groups compared to baseline measures. Vitamin C did not offer a therapeutic effect. No significant difference between group plasma levels post 1-month.

## Discussion

**Fig. 2:**
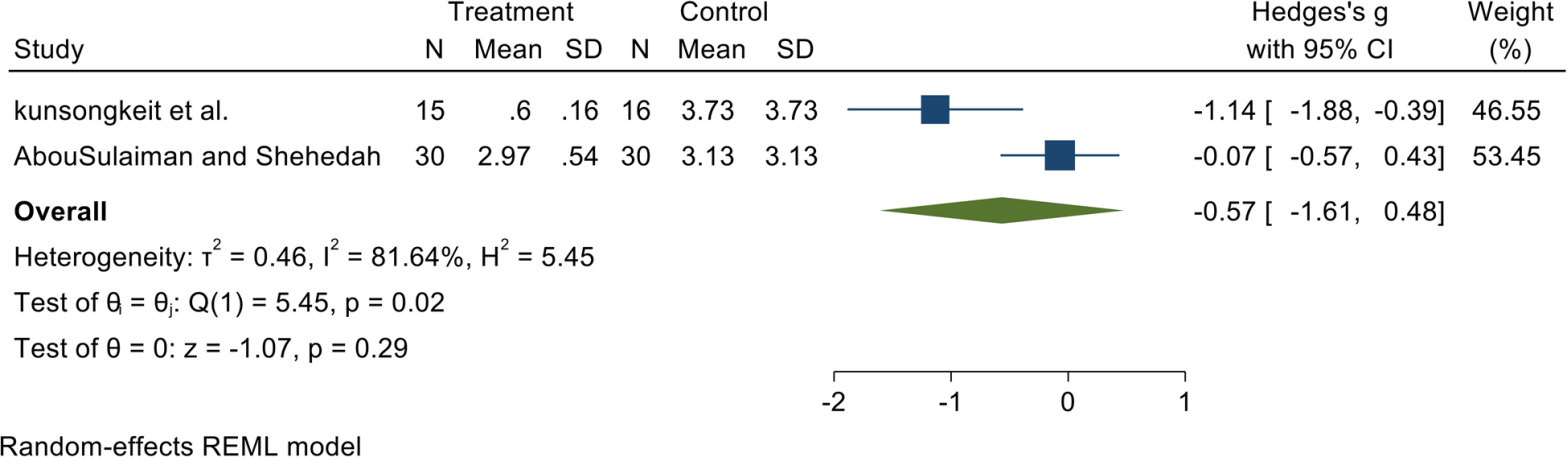
Forest plot presenting 1-month post-therapy probing depth by comparing vitamin C intake vs. control

**Fig. 3:**
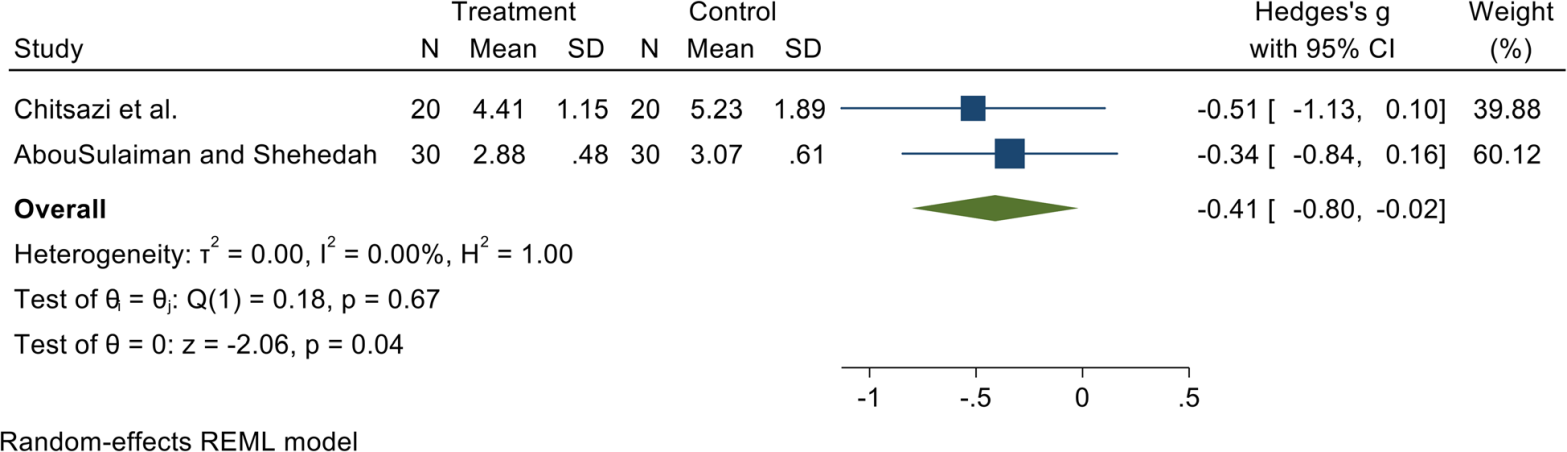
Forest plot presenting 3-month post-therapy probing depth by comparing vitamin C intake vs. control

In the present review, we found that administration of vitamin C as an adjunct to non-surgical periodontal therapy did not result in clinically significant improvements in pocket probing depths at 3 months in patients with periodontitis (Figs. 2 and 3). Numerous literature reviews have been published previously that identified the relationship between serum concentration of vitamin C and periodontitis using evidence from cross-sectional and case-control studies. Since interventional studies including randomized controlled trials are the “gold standard” in the hierarchy of observational studies, we accumulated randomized controlled trial-based evidence, using an exhaustive search strategy to establish if a relationship exists between vitamin C supplementation and periodontal status of subjects following non-surgical periodontal therapy.

Scaling and root planning (SRP) is the first-line approach for the treatment of periodontitis cases. It has remained the “gold standard” of periodontal therapy for decades and is usually quite predictable [23]. Over the years, research has been focused on finding anti-inflammatory, chemotherapeutic, and host-modulatory agents that can significantly enhance the results of SRP. It has been demonstrated that SRP alone is capable of reducing oxidative stress and restoring redox balance until 3 months post SRP but not at 6 months despite thorough oral hygiene maintenance implying that there is a decreased ability of periodontal tissues against ROS activity in periodontitis patients even after successful non-surgical therapy [24]. Moreover, there is sufficient evidence to show that supplemental treatment with antioxidants like vitamin E, taurine, and lycopene resulted in improved clinical periodontal parameters, increased activities of local and systemic antioxidants, and reduced levels of local and systemic reactive oxygen species (ROS) when compared with non-surgical periodontal therapy alone [25].These findings taken together support the need for supplementation of antioxidants in addition to SRP not only to improve short-term clinical outcomes but also to maintain redox balance and normalize oxidant stress after SRP over the long-term(> 6 months). Micronutrients, such as vitamin C, possess potent antioxidant [26], immune-modulatory [27], and angiogenic properties [28] making it a promising agent for adjunctive use with initial periodontal therapy. However, evidence regarding its efficacy as an adjunct to SRP is either conflicting or unavailable to warrant its adoption in routine therapy.

A total of six RCTs which were included in this review, vitamin C was administered in conjunction with SRP in varying modes and doses in adult participants > 23 years of age. The minimum follow-up period ranged from 14 days to 6 months, with four studies mandating at least a 3-month follow-up [17–19, 21]. Five out of six included studies found that vitamin C supplementation was associated with improved periodontal outcome measures of PI, SBI, GI, PPD, and CAL. Supplemental administration of vitamin C along with initial therapy did improve indices of gingival bleeding and inflammation in test groups comprising of gingivitis patients [20, 21] and diabetics [20] but such a relationship was not observed in periodontitis group. The reduction in gingival bleeding and inflammation is expected in gingivitis cases and there is evidence from 2 experimental gingivitis studies [29, 30] which demonstrate an inverse relationship between vitamin C supplementation and gingivitis.

In periodontitis, such a relationship with vitamin C has not been observed probably because periodontitis is a distinct condition from gingivitis [12] and also there may be factors limiting its action in periodontitis tissues [31]; therefore, supplementation with vitamin C may not exhibit the same effect as observed in gingivitis. Despite the ability of ascorbic acid to induce progenitor cells of the periodontal tissues to differentiate and promote wound healing in vitro [9], there remains no evidence suggesting that it has the potential to result in the gain of clinical attachment in vivo. This may be another substantial reason that does not support its additional benefit in reducing PPD in the long-term for severe periodontitis [9]. Vitamin C did, however, have a preventative beneficial effect on the maintenance of periodontal health, and the concentration of vitamin C in serum/plasma was found to be associated with periodontal health in our review.

Antioxidants rarely act alone in vivo and always in an orchestrated manner [32]. Consumption of citrus fruits is more effective in raising the plasma vitamin C levels when compared to the administration of high-dose supplements [33]. Also, studies with dietary intervention in the form of grape fruits [34] and kiwi fruits [35] have shown to raise plasma levels of vitamin C over recommended daily allowance. Besides, these natural sources are rich in other micro and phytonutrients such as flavonoids which may act as co-adjuvants. In that case, would it be justified to persist with trials employing single vitamin antioxidants from synthetic sources? Hence, our approach to adjunctive therapy needs to be reconsidered in light of these recent findings. Perhaps a more holistic, diet-based interventional approach with whole foods may be the new direction for future research in this area.

### Limitations and future implications

Since the outcome measures varied in the studies that were included in this review, this led to heterogeneous data generation and comparisons were made within the results of the studies reviewed. Also, the number of articles included in this study was limited. Larger, well-controlled clinical studies are the need of the hour to build upon the currently limited evidence along with more interventional studies to assess the role of systemic and risk factors such as diabetes and smoking with periodontitis and vitamin C.

## Conclusion

Administration of vitamin C as an adjunct to non-surgical periodontal therapy did not result in clinically significant improvements in pocket probing depths at 3 months in periodontitis patients. With the limited evidence available, no recommendation can be made for supplementation of vitamin C in conjunction with initial periodontal therapy for subjects with periodontitis to improve primary treatment outcome measures. More long-term, well-designed, longitudinal studies with better assessment criteria are needed to produce conclusive evidence on the subject.

## Data Availability

All data generated or analyzed during this study are included in this article.

## Abbreviations

ALA: Alpha-linolenic acid
BOP: Bleeding on probing
CAL: Clinical attachment level
CASP: Critical Appraisal Skills Programme
CI: Confidence interval
DHA: Docosahexaenoic acid
EPA: Eicosapentaenoic acid
GI: Gingival index
GSI: Gingivitis severity index
NSAIDs: Non-steroidal anti-inflammatory drugs
NSPT: Non-surgical periodontal therapy
PI: Plaque index
PMNs: Polymorphonuclear leukocytes
PPD: Periodontal/probing pocket depth
RCTs: Randomized control trials
PRISMA: Preferred Reporting Items for Systematic Reviews and Meta-Analyses
RoB-2: Risk of Bias Tool 2 for randomized trials
ROS: Reactive oxygen species
SBI: Sulcus Bleeding Index
SD: Standard deviation
SMD: Standardized mean difference
SRP: Scaling and root planning
TAOC: Total antioxidant capacity
